# Usefulness of Background Coloration in Detection of Esophago-Pharyngeal Lesions Using NBI Magnification

**DOI:** 10.1155/2012/529782

**Published:** 2012-08-15

**Authors:** Hitomi Minami, Haruhiro Inoue, Haruo Ikeda, Hitoshi Satodate, Shigeharu Hamatani, Kazuhiko Nakao, Shin-ei Kudo

**Affiliations:** ^1^Digestive Disease Center, Showa University Northern Yokohama Hospital, 35-1, Chigasaki-Chuo, Tsuzuki, Yokohama 224-8503, Japan; ^2^Department of Gastroenterology and Hepatology, Nagasaki University, 1-7-1, Sakamoto, Nagasaki, Japan

## Abstract

*Background and Aim*. We evaluated the usefulness of background coloration (BC), a color change in the area between intrapapillary capillary loops (IPCLs) in the early esophago-pharyngeal lesions using NBI with magnificaiton. *Methods*. Between April 2004 and March 2010, a total of 294 esophago-pharyngeal lesions were examined using NBI with magnification, and the presence of BC and IPCL patterns were assessed. Using BC, discrimination of squamous cell carcinoma (SCC) or high-grade neoplasia (HGN) from low-grade neoplasia (LGN) or nonatypia was conducted. *Results*. Among 294 lesions, 209 lesions (71.1%) were positive for BC, while 85 (28.9%) were negative. In the BC-positive group, 187 lesions (89.5%) were diagnosed as SCC/HGN. And 68 lesions (80.0%) in the BC-negative group were diagnosed as LGN/nonatypia. Overall accuracy of BC to discriminate SCC/HGN from LGN/nonatypia was 87.3%. The sensitivity and specificity were 91.9%, 76.7%. BC could discriminate SCC/HGN from LGN/nonatypia accurately (*P* < 0.0001). Among 68 lesions classified into the IPCL type IV, the BC-positive group (*n* = 26) included 21 SCC/HGN lesions, while there were 36 LGN/nonatypia lesions in the 42 BC-negative lesions. *Conclusions*. BC is a useful finding in differentiating SCC/HGN from LGN/nonatypia lesions in the esophagus especially when it is combined with IPCL pattern classification.

## 1. Introduction

**Figure 1:**
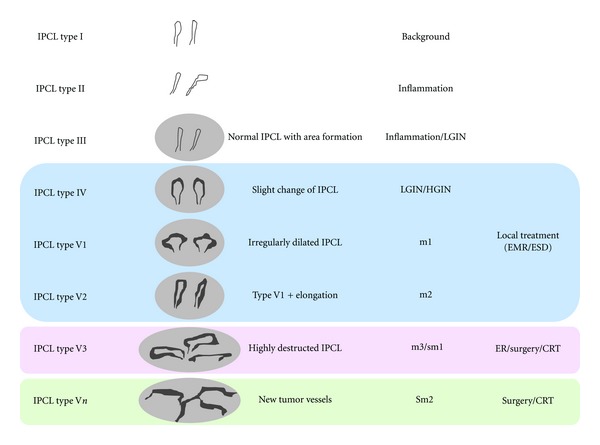
IPCL pattern classification, which was introduced by Inoue et al. in 2001. Most of IPCL type I to III is corresponding to benign pathology including inflammation and low grade neoplasia (LGN). Approximately 50% of IPCL type IV is corresponding to LGN, and the rest is corresponding to malignant pathology including high grade neoplasia (HGN). IPCL type V is a malignant pattern and subclassified into 4 categories. V1 and V2 could be removed by endoscopic resection (ER) such as EMR and ESD. IPCL type V3 includes m3 and sm1 cancers. IPCL type Vn is a pattern for massively submucosally invasive cancers. *CRT: chemoradiotherapy.

The usefulness of narrowband imaging (NBI) magnification in detecting early esophago-pharyngeal lesions including noninvasive high grade neoplasia and invasive squamous cell carcinoma (SCC) has already been reported [1, 2]. Using NBI, such esophago-pharyngeal lesions are recognized as brown-colored area. Magnified endoscopic observation with NBI has been performed to visualize intrapapillary capillary loops (IPCLs) [3–5], the superficial microvascular architecture in the mucosa covered with squamous epithelia.  Figure 1 shows IPCL pattern classification, which was introduced by Inoue in 2001. The IPCL pattern is useful in the diagnosis of the depth of tumor invasion for esophago-pharyngeal squamous cell neoplasms. Nevertheless, distinguishing malignant from noncancerous lesions is sometimes difficult even for experienced endoscopists.

We have found that background coloration (BC), a color change in the area between IPCL, is seen in the esophago-pharyngeal neoplastic lesions but not in the benign lesions using NBI with magnifying endoscopy. Therefore, we sought to determine whether BC could discriminate SCC or high-grade neoplasia (HGN) from low-grade neoplasia (LGN) or nonatypia.

## 2. Methods

### 2.1. Patients

Three hundred and seventy lesions, which were found between April 2004 and March 2010 in our institution, were enrolled in this study. One lesion without pathology and 53 lesions without detailed magnifying observation of IPCL pattern were excluded. And 7 cases were excluded because the margin of the brownish area was difficult to be identified (IPCL type II). In total, two hundred and twenty-two patients (male : female = 169 : 53) aged 67.0 ± 16.0 y.o. (range 45–92 y.o., median 68.5 y.o.) with 294 lesions were enrolled in this study. Two hundred and thirty lesions were treated by endoscopic mucosal resection/endoscopic submucosal dissection (EMR/ESD), and 64 lesions were pathologically confirmed by biopsy. The mean diameter of the lesions was 15.3 mm (range 1–95 mm, median 10 mm).

All samples were obtained with the written informed consent of the patients prior to their inclusion, in accordance with the Helsinki Declaration and under approval by the Showa University Ethics Committee.

### 2.2. Endoscopic Examination

We used GIF-H260Z (Olympus medical systems Co., Tokyo, Japan) comprising a high-resolution white-light video endoscope equipped with NBI system. An NBI system has a high-resolution mode in which the white light image (WLI) is composed of sequential images taken through red, green, and blue (RGB) bandpass filters. In NBI mode, the central wavelengths of the RGB filters (445 nm (blue), 540 nm (green), and 620 nm (red)) are narrowed to 415 nm (blue) and 540 nm (green). Using the wavelength dependence of the light penetration depth into the mucosa and the hemoglobin absorption characteristics in the surface layer, NBI technology allows the mucosal surface layer to be displayed in high contrast and enhances hemoglobin-rich areas such as blood vessels.

Patients underwent upper endoscopy under conscious sedation with intravenous pethidine hydrochloride (35–70 mg, Mitsubishi Tanabe Pharmaceutical Co., Osaka, Japan) supplemented with Diazepam (5–10 mg, Takeda Pharmaceutical Co., Osaka, Japan). In order to suppress esophageal peristalsis, either scopolamine butylbromide (20 mg, Boehringer Ingelheim GmbH, Ingelheim, Germany) or glucagon (1-2 mg, Novo Nordisk, Bagsværd, Denmark) was also administered intravenously. All procedures were carried out by two experienced endoscopists (M.H. and H.I.).

After a brownish area was found with NBI, the lesion was observed with NBI magnification to be classified into IPCL pattern classification that has been introduced by Inoue et al. IPCL pattern classification has been reported to be a useful tool to discriminate cancer and dysplasia from benign lesions. Inoue classified esophageal lesions into five categories depending on the irregularity of the IPCL. IPCL pattern classification has five categories, and each of them is corresponding to the pathology from benign to squamous cell carcinoma.

**Figure 2:**
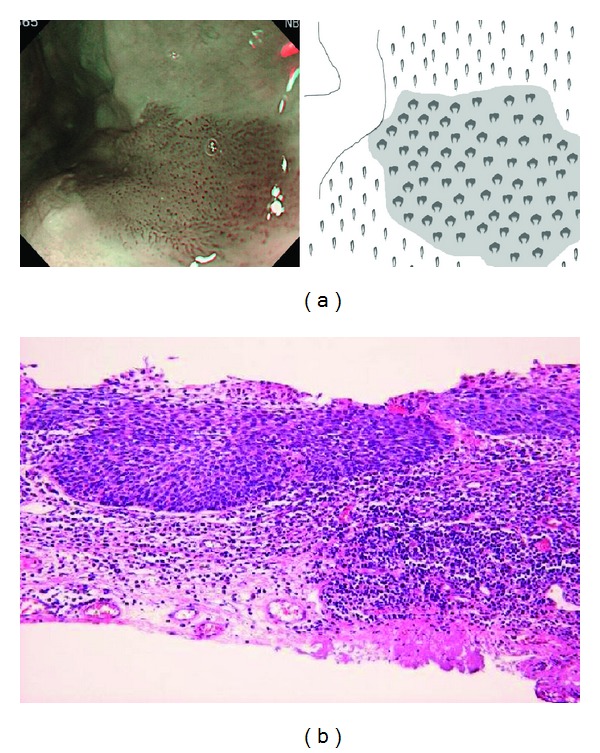
(a) Positive for background coloration (left panel). Color of the space between each dilated IPCL is brownish compared to normal IPCL area, as pointed out in schema (right image). (b) Endoscopic resection was carried out. Pathological result was T1a-LPM squamous cell carcinoma (squamous cell carcinoma, moderately differentiated type, pT1a-LPM, INFb, ly0, v0, pHM0, pVM0).

**Figure 3:**
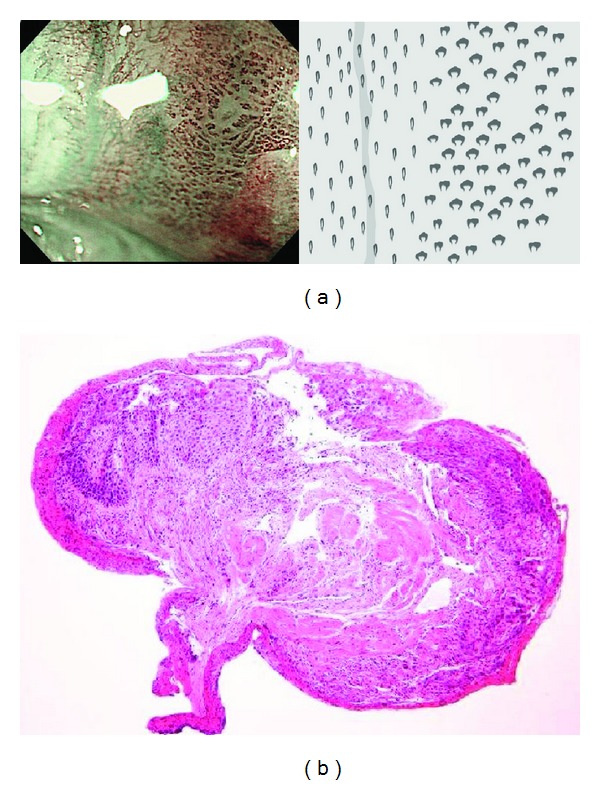
(a) Negative for background coloration (left panel). The color of the area between dilated IPCL is same to surrounding normal IPCL area as pointed out in schema (right image). (b) The lesion was pathologically confirmed as inflamed mucosa by biopsy.

Then, the lesion was observed with NBI magnification, and BC was evaluated. If there is a color change in the area between IPCLs, the lesion was regarded as being positive for BC (Figures 2(a) and 2(b)). If there is no color change in the area, the lesion was regarded as being negative for BC (Figures 3(a) and 3(b)). For larger lesions with heterogeneous BC findings, we categorized the lesion into BC positive group if BC-positivity was clearly observed at least in one part of the lesion.

### 2.3. Histological Evaluation

Each specimen resected by EMR/ESD was fixed in 10% formalin and embedded in paraffin wax. The tissue specimens were cut at a thickness of 2 mm, and all sections were routinely evaluated for pathological diagnosis. Biopsy specimens collected by endoscopists on suspicion of or to rule out SCC were also used to examine diagnostic histological findings. All specimens were evaluated by experienced gastrointestinal pathologists. Histology was assessed according to the following four categories of Vienna classification: invasive SCC, HGN, low-grade neoplasia (LGN), and negative or indefinite for neoplasia (nonatypia).

### 2.4. Statistical Analysis

Sensitivity, specificity, positive predictive value, negative predictive value, and overall accuracy of identifying HGN or SCC were estimated for BC and IPCL pattern classification, using histological findings as the gold standard. *P *values <0.05 were considered to be statistically significant. Data were analyzed using Statview Version 5.0 (SAS Institute Co., Cary, NC).

## 3. Results

Among 294 lesions, 209 lesions (71.1%) were positive for BC and 85 lesions (28.9%) were negative. As shown in Table 1, most invasive SCC (99.1%) and the majority of HGN (82.2%) showed BC positivity. Thus, 91.6% of HGN/invasive SCC was diagnosed positive for BC. On the other hand, 68 lesions (80.2%) in the BC-negative group were nonatypia (mostly inflammatory change) or LGN. Overall accuracy of BC for discrimination of HGN and invasive SCC was 87.3%. Sensitivity, specificity, positive predictive value, and negative predictive value were 91.9%, 76.7%, 90.1%, and 80.2%, respectively.

Table 2 shows correlation between tumor invasion depth and BC positivity. There was significant increase in positive rates of BC in proportion to the tumor invasion depth. On the contrary, BC negative rates were decreased in association with the tumor infiltration depth.

It has been reported that IPCL type III is a typical pattern for benign lesions including nonatypia and LGN, while the type V is highly suggestive of SCC in Japanese literature. In line with this, we confirmed that the type III and V could clearly discriminate invasive SCC/HGN from LGN/nonatypia. In contrast, type IV was inconclusive, as lesions classified into this IPCL pattern included substantial numbers of HGN/SCC and LGN/nonatypia. Lesions with IPCL type III, which usually represent inflammation or LGN, can be carefully observed. IPCL type V which is corresponding to malignancy obviously requires some treatment including EMR/ESD. However, IPCL type IV is still inconclusive because more than half of them are nonmalignant. We propose that IPCL type IV lesions should be considered for pathological confirmation. How to make more reliable endoscopic diagnosis or whether to remove or not has been a problem of great interest.

Focusing on IPCL pattern IV, 41 of 68 lesions showed BC negativity, and 36 (85.7%) of them were pathologically confirmed as nonatypia or LGN. On the other hand, 22 (81.5%) of 27 lesions, which were diagnosed as BC positive, were pathologically HGN or SCC (Table 3). In this study, overall accuracy of BC in lesions showing IPCL type IV was no less than 85.3%. Similarly to the IPCL type V group, statistically significant interrelation between BC and pathology was seen (*P* < 0.0001). The accuracy of IPCL pattern IV was significantly improved when it was combined with BC (Table 4). Therefore, BC added significant improvement in diagnostic ability of IPCL classification.

**Figure 4:**
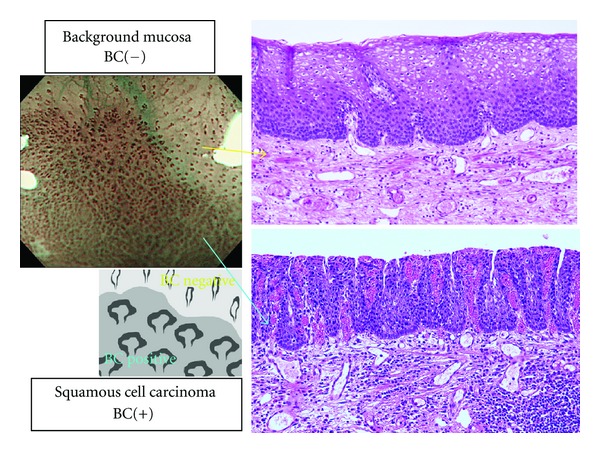
To evaluate the pathological difference between background mucosa (BC negative) and cancerous area (BC positive), we compared two lesions that were positive for BC and negative for it. See the differences in number of cells, N/C ratio, state of keratosis, level of inflammatory cells infiltration.

In order to evaluate the cause of BC, we compared two parts of the same lesion, which was positive for BC and negative for it (Figure 4). Histopathologically some differences were seen in number of cells, N/C ratio, thickness of stratum corneum layer, and level of inflammatory cells infiltration.

## 4. Discussion

Iodine staining has been utilized to detect esophageal squamous cell carcinoma as the most reliable diagnostic tool [6, 7]. Previously reported data shows that the diagnostic accuracy of differentiating SCC from all the Lugol voiding lesions was 13.0 to 33% [8, 9]. Ohmori et al. reported that pinkish color change few minutes after spraying iodine is useful to differentiate SCC from benign changes [10, 11]. However, iodine staining has certain unfavorable aspects such as prolongation of the procedure time, iodine allergy in some patients, and uncomfortable side effects including chest discomfort and cough. It is therefore difficult to apply iodine staining to all patients other than high-risk populations such as patients with a history of head and neck cancer, heavy smokers, and alcohol abusers. Especially in the oro-pharyngeal region, chromoendoscopy with iodine staining cannot be easily applied because of these uncomfortable effects.

On the other hand, NBI system is easily applied by pushing a button on the endoscope without efforts of spraying iodine or waiting for the color change. Takenaka and Kuraoka et al. compared NBI with chromoendoscopy with iodine staining in detection for esophageal SCC or HGN [12, 13]. They reported that NBI was significantly superior to the chromoendoscopy in the overall accuracy of detection. Also Muto et al. reported that NBI combined with magnifying endoscopy significantly improved the detection rates for SCC with quite high sensitivity [14]. However, specificity for esophageal region was 42.1% that still is not sufficient. Kuraoka assessed the reason of this relatively low specificity as hypervascularity due to inflammation and improper keratinization [12].

Inoue et al. already reported IPCL pattern classification as an effective diagnostic tool in detecting squamous cell carcinoma [3–5]. Overall accuracy, sensitivity, specificity, positive predictive value, and negative predictive value have been reported to be 82.9%, 97.3%, 66.2%, 77.0%, and 95.4%. IPCL type I, II, and III are corresponding to benign pathology including LGN. On the other hand, IPCL type IV and V are expected to represent malignancy including HGN. They have reported that diagnostic accuracy of IPCL type III and type V is 94.8% and 89.4%, which is sufficient, respectively. However, more than 50% of the lesions that were diagnosed as IPCL type IV were pathologically proved to be benign. This means that there are some risks of overdiagnossis, possibly leading to overtreatment.

In contrast, assessment using BC resulted in high sensitivity and specificity for distinguishing between HGN/SCC and LGN/nonatypia. Moreover, focusing on IPCL type IV, BC added significant improvement in diagnostic ability of IPCL classification.

It is beyond the scope of this study to explore what BC represents. There were some histopathological differences in number of cells, N/C ratio, thickness of stratum corneum layer, and level of inflammatory cells infiltration between the BC-positive and the surrounding BC-negative one as shown in Figure 3. Fujii et al. evaluated the correlation between density of microvessels and pathology [15]. They reported that microvascular density increases step by step from basal cell hyperplasia through dysplasia to carcinoma. These findings may explain in part the BC significance. Also NBI image mechanically reflects the wavelength that is specific to hemoglobin. This means that the cause of BC potentially carries another possibility such as existence of red blood cells in intra- or extraepithelium. The cause of this phenomenon is still quite uncertain, and there are many possibilities. And clearly, further studies are needed to illuminate this issue. This is a single center prospective study. There should also be needed further prospective study to elucidate the limitations such as intra- and interobserver variations.

In conclusion, BC positivity seems to be a useful and reliable finding for suggestive malignant lesions in the esopahgo-pharyngeal area. This finding has to be solidified by measuring intra- and interobserver variation. In combination with IPCL pattern classification, BC can provide additional information on accurate discrimination of SCC/HGN from LGN/nonatypia.

## Figures and Tables

**Table 1:** Histological diagnosis for 294 esophago-pharyngeal lesions. More than 90% of BC positive lesions were histologically confirmed as HGIN or SCC. On the other hand, 80.0% of BC negative group were diagnosed as noncancer, *P* < 0.0001.

	BC positive (209 lesions)	BC negative (85 lesions)
Invasive squamous cell carcinoma	113 (99.1%)	1 (0.9%)
HGIN	74 (82.2%)	16 (17.8%)
LGIN	7 (20.6%)	27 (79.4%)
Nonatypia	15 (26.8%)	41 (73.2%)

**Table 2:** Significant correlation was seen between tumor depth and presence of BC, *P* < 0.0001.

	LGN/nonatypia	HGN/T1a-EP	T1a-LPM	T1a-MM/T1b-SM
BC (+)	22	74	70	43
	(24.4%)	(82.2%)	(98.6%)	(100%)
BC (−)	68	16	1	0
	(75.6%)	(17.8%)	(1.4%)	(0%)

Total	90	90	71	43

**Table 3:** Relation between IPCL pattern classification and pathology, *P* < 0.0001.

	Nonatypia/LGIN	HGIN/invasive SCC
IPCL type III	39 (95.1%)	2 (4.9%)
IPCL type IV	41 (60.3%)	27 (39.7%)
IPCL type V	10 (5.4%)	175 (94.6%)

**Table 4:** BC pairing with IPCL type IV significantly improved the diagnostic accuracy in detecting squamous cell carcinoma including HGN.

IPCL type IV 68 lesions	BC (+)	27	LGIN	5	*P* < 0.0001
			HGIN/SCC	22	
	BC (−)	41	LGIN	36	
			HGIN/SCC	5	

## References

[1] Gono K, Obi T, Yamaguchi M (2004). Appearance of enhanced tissue features in narrow-band endoscopic imaging. *Journal of Biomedical Optics*.

[2] Yoshida T, Inoue H, Usui S, Satodate H, Fukami N, Kudo SE (2004). Narrow-band imaging system with magnifying endoscopy for superficial esophageal lesions. *Gastrointestinal Endoscopy*.

[3] Inoue H (2007). Endoscopic diagnosis of tissue atypism (EA) in the pharyngeal and esophageal squamous epithelium; IPCL pattern classification and ECA classification. *Kyobu Geka*.

[4] Inoue H (2007). Magnifying endoscopic diagnosis of tissue atypia and cancer invasion depth in the area of pharyngo-esophageal squamous epithelium by NBI enhanced magnification image: IPCL pattern classification. *Advanced Digestive Endoscopy*.

[5] Kumagai Y, Inoue H, Nagai K, Kawano T, Iwai T (2002). Magnifying endoscopy, stereoscopic microscopy, and the microvascular architecture of superficial esophageal carcinoma. *Endoscopy*.

[6] Shiozaki H, Tahara H, Kobayashi K, Yano H, Tamura S, Imamoto H (1990). Endoscopic screening of early esophageal cancer with the Lugol dye method in patients with head and neck cancers. *Cancer*.

[7] Kouzu T, Suzuki Y, Yoshimura S, Yoshimura N, Hishikawa E, Arima M (1998). Feature of screening-detected cancer and progress of treatment—esophageal cancer. *Gan To Kagaku Ryoho*.

[8] Shimizu Y, Tukagoshi H, Fujita M, Hosokawa M, Kato M, Asaka M (2001). Endoscopic screening for early esophageal cancer by iodine staining in patients with other current or prior primary cancers. *Gastrointestinal Endoscopy*.

[9] Yokoyama A, Ohmori T, Makuuchi H, Maruyama K, Okuyama K, Takahashi H (1995). Successful screening for early esophageal cancer in alcoholics using endoscopy and mucosa iodine staining. *Cancer*.

[10] Ohmori T, Yokoyama A (2001). Clinical usefulness of pink-color sign. *Gastroenterological Endoscopy*.

[11] Shimizu Y, Omori T, Yokoyama A (2008). Endoscopic diagnosis of early squamous neoplasia of the esophagus with iodine staining: high-grade intra-epithelial neoplasia turns pink within a few minutes. *Journal of Gastroenterology and Hepatology*.

[12] Takenaka R, Kawahara Y, Okada H (2009). Narrow-band imaging provides reliable screening for esophageal malignancy in patients with head and neck cancers. *American Journal of Gastroenterology*.

[13] Kuraoka K, Hoshino E, Tsuchida T, Fujisaki J, Takahashi H, Fujita R (2009). Early esophageal cancer can be detected by screening endoscopy assisted with Narrow-band Imaging (NBI). *Hepato-Gastroenterology*.

[14] Muto M, Minashi K, Yano T (2010). Early detection of superficial squamous cell carcinoma in the head and neck region and esophagus by narrow band imaging: a multicenter randomized controlled trial. *Journal of Clinical Oncology*.

[15] Fujii S, Yamazaki M, Muto M, Ochiai A (2010). Microvascular irregularities are associated with composition of squamous epithelial lesions and correlate with subepithelial invasion of superficial-type pharyngeal squamous cell carcinoma. *Histopathology*.

